# Retraction: A Novel Triterpenoid Isolated from the Root Bark of *Ailanthus excelsa* Roxb (Tree of Heaven), AECHL-1 as a Potential Anti-Cancer Agent

**DOI:** 10.1371/journal.pone.0231249

**Published:** 2020-03-27

**Authors:** The PLOS ONE Editors

After this article [1] was published, concerns were raised about the mouse tumor sizes reported in Fig 6. The figure reports tumor volume in percent growth compared to baseline, but the authors provided actual tumor size data in post-publication discussions. These data confirmed that tumor sizes up to 13.12 cm^3^ were observed in the study.

The authors commented that per their observations mouse mobility was not hindered during the experiments, and that extra care was taken to make food easily available. They noted that they observed animals twice daily for morbidity and mortality, and animals were assessed once daily (after treatment) for signs of behavioral changes, food and water consumption, body weight, illness, and reactions to treatment. Per the study protocol, animals were to be euthanized if they were determined to be in overt pain and/or distress, if death seemed imminent, or if they appeared beyond the point where recovery appears possible.

The authors provided a copy of an ethics approval document per the journal’s request, and none of the article’s authors were included in the list of individuals authorized to conduct procedures under the approved proposal.

A member of *PLOS ONE*’s Animal Research Advisory Group assessed the article and the authors’ comments and confirmed that the tumor sizes reported far exceed community standards for humane endpoint limits in mouse tumor studies. The advisor noted that the tumor results present a significant animal welfare and ethical concern.

In light of the above concerns, the *PLOS ONE* Editors retract this article. The editors regret that these concerns were not addressed at the time of the original review process.

The article’s authors either could not be reached or did not respond directly to the retraction notification.
